# Data integration from two microarray platforms identifies bi-allelic genetic inactivation of *RIC8A *in a breast cancer cell line

**DOI:** 10.1186/1755-8794-2-26

**Published:** 2009-05-11

**Authors:** Aslaug Aamodt Muggerud, Henrik Edgren, Maija Wolf, Kristine Kleivi, Emelyne Dejeux, Jörg Tost, Therese Sørlie, Olli Kallioniemi

**Affiliations:** 1 Department of Genetics, Institute for Cancer Research, Norwegian Radium Hospital, Oslo University Hospital, 0310 Oslo, Norway; 2 Faculty of Medicine, Division The Norwegian Radium Hospital, University of Oslo, 0316 Oslo, Norway; 3 Institute for Molecular Medicine (FIMM), University of Helsinki, Biomedicum Helsinki 2 U, Tukholmankatu 8, FIN-00290 Helsinki, Finland; 4 Medical Biotechnology, VTT Technical Research Centre of Finland and Centre for Biotechnology, University of Turku, FIN-20520 Turku, Finland; 5 CEA-Institut de Génomique, Centre National de Génotypage, Laboratory for Epigenetics, 2 rue Gaston Crèmieux, 91000 Evry, France; 6 Department of Informatics, University of Oslo, Oslo, Norway

## Abstract

**Background:**

Using array comparative genomic hybridization (aCGH), a large number of deleted genomic regions have been identified in human cancers. However, subsequent efforts to identify target genes selected for inactivation in these regions have often been challenging.

**Methods:**

We integrated here genome-wide copy number data with gene expression data and non-sense mediated mRNA decay rates in breast cancer cell lines to prioritize gene candidates that are likely to be tumour suppressor genes inactivated by bi-allelic genetic events. The candidates were sequenced to identify potential mutations.

**Results:**

This integrated genomic approach led to the identification of *RIC8A *at 11p15 as a putative candidate target gene for the genomic deletion in the ZR-75-1 breast cancer cell line. We identified a truncating mutation in this cell line, leading to loss of expression and rapid decay of the transcript. We screened 127 breast cancers for *RIC8A *mutations, but did not find any pathogenic mutations. No promoter hypermethylation in these tumours was detected either. However, analysis of gene expression data from breast tumours identified a small group of aggressive tumours that displayed low levels of *RIC8A *transcripts. qRT-PCR analysis of 38 breast tumours showed a strong association between low *RIC8A *expression and the presence of *TP53 *mutations (P = 0.006).

**Conclusion:**

We demonstrate a data integration strategy leading to the identification of *RIC8A *as a gene undergoing a classical double-hit genetic inactivation in a breast cancer cell line, as well as *in vivo *evidence of loss of *RIC8A *expression in a subgroup of aggressive *TP53 *mutant breast cancers.

## Background

**Figure 1:**
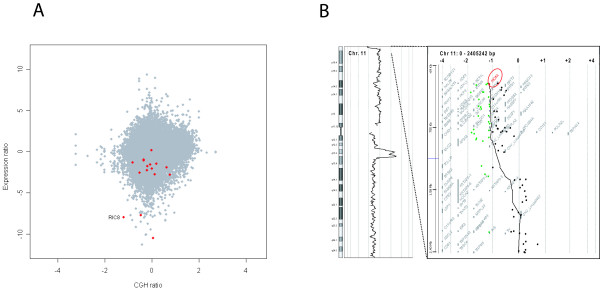
**Overview of the microarray data integration**. A)"DNA/RNA outlier plot" of cell line ZR-75-1. The x-axis indicates the DNA copy number ratio of all 54675 probe sets on the Affymetrix U133plus2.0 array. These ratios were calculated from Agilent array CGH data for the same genes. The y-axis indicates the expression ratio of all probe sets compared to the median of other cell lines. Putative NMD hits are shown in red. *RIC8A *is both heterozygously deleted, underexpressed compared to the other cell lines and its expression was induced by emetine treatment. B)A schematic view of chromosome 11 in ZR-75-1, as well as the deleted region at 11p15.5. *RIC8A *is located ~200 kb from the p-telomere.

Identification of genetic events that are involved in the development of human cancer has been facilitated by the availability of advanced genomic microarray platforms [1]. However, often the mechanisms by which these genetic events influence target genes in the chromosomal regions affected and eventually on the tumourigenic process, remain unknown. Altered genomic regions identified by e.g. array-based comparative genomic hybridization (aCGH), may contain hundreds of candidate genes making the identification of specific disease-causing changes challenging. We have recently applied nonsense-mediated mRNA decay (NMD) microarray [2], and integrated data from this technology with data from aCGH [3,4]. Transcripts with nonsense mutations are selectively degraded by the NMD pathway during translation [5]. Inhibition of the NMD pathway can be exploited to detect changes in the stability of transcripts carrying such truncating mutations. As bi-allelic inactivation of tumour suppressor genes often involves loss of the second copy of the genomic region harbouring these genes, integrating data from using NMD microarrays with whole genome CGH microarray data may help in order to focus the search to deleted chromosomal areas (Figure 1). Using integration of unbiased genome-wide data from these two technologies, we describe here the identification of a putative target gene, *RIC8A*, located in a frequently deleted region at 11p15 in breast cancer. *RIC8A *was flagged as a candidate for undergoing NMD in the breast cancer cell line ZR-75-1. Furthermore, we bioinformatically explored the *in vivo *levels of expression of this gene in datasets from two published breast cancer studies [6,7]. *RIC8A *interacts with GDP-bound G-alpha protein [8], has been found to be associated with mitotic spindle alignment in asymmetric cell division in the fly [9], and includes a protein motif that is involved in regulation of mitosis [10]. We therefore hypothesized that this gene may play a role in breast cancer development or progression.

## Methods

### Cell culture and emetine treatment

We used six commonly studied breast cancer cell lines, representing different types of breast cancer; MDA-MB-468 (ER-, PR-, *TP53 *mutation), MDA-MB-231 (ER-, PR-, *TP53 *mutation), ZR-75-1 (ER+, PR+), MCF7 (ER+, PR+), BT-474 (ER+, PR+, *TP53 *mutation) and T-47D (ER+, PR+, *TP53 *mutation) and three non-malignant cell lines; HMECs (non-malignant human mammary epithelial cells), IMR90 (normal lung fibroblasts) and WS1 (normal skin fibroblasts). All cell lines were obtained from American Type Culture Collection and grown in accordance with the distributor's instructions. Both malignant and non-malignant cell lines were treated with the translation inhibitor emetine dihydrochloride hydrate (Sigma-Aldrich, St Louis, MO). For each cell line, parallel cell cultures were grown in 175 cm^2 ^flasks until 70–80% confluence. Half of the subconfluent cultures were treated with 100 μg ml^-1 ^of emetine dihydrochloride hydrate while the other half were left as untreated controls. Both emetine-treated and untreated cells were incubated at 37°C for 10 h. After drug exposure, cells were trypsinated, cell pellets snap-frozen and total RNA was extracted using RNeasy mini kit (Qiagen, GmbH, Hilden Germany) as described by the manufacturer. RNA quality was evaluated using an Agilent 2100 Bioanalyzer (Agilent Technologies, Palo Alto, California, USA). Genomic DNA was extracted from untreated cells using the NucleoSpin Tissue kit with the protocol for cultured cells (Macherey-Nagel, Düren, Germany).

### NMD microarrays

We used Affymetrix Human Genome U133 plus 2.0 GeneChip oligonucleotide arrays to compare emetine-treated and untreated cells. 3 μg of total RNA per sample was prepared according to the manufacturer's protocols. For hybridization, fragmented cRNA (15 μg from each sample) was used. The arrays were stained with phycoerythrin-streptavidin and the signal intensity was amplified by treatment with a biotin-conjugated anti-streptavidin antibody, followed by a second staining using phycoerythrin-streptavidin. Finally, the stained arrays were scanned using the GeneChip Scanner 3000 (Affymetrix). Data were analyzed using GeneChip Operating Software 1.1 (Affymetrix).

### aCGH analysis

Array-based CGH was carried out using DNA from the untreated breast cancer cell lines. Agilent Human Genome CGH microarray 44 k was utilized according to the protocol provided by the manufacturer (Agilent Technologies, Palo Alto, California, USA). Genomic DNA pooled from healthy female donors was used as reference in all hybridizations. Briefly, six micrograms of digested and purified tumour and reference DNA was labelled with Cy5-dUTP and Cy3-dUTP (Perkin-Elmer, Wellesley, Massachusetts, USA), respectively, in a random priming reaction using BioPrime DNA Labelling System (Invitrogen, Carlsbad, California, USA). Labelled tumour and reference samples were pooled and hybridized onto the arrays according to the protocol. After hybridization arrays were washed and scanned with a laser scanner (G2565 Scanner, Agilent Technologies). Feature Extraction software version 7.5.1 (Agilent Technologies) was used to extract the signal intensities. CGH Analytics software version 3.4.27 (Agilent Technologies) was used for data visualization.

### Microarray data analysis

Data from the Affymetrix gene expression arrays were normalized using the dChip method [11]. Gene expression and aCGH data were combined by calculating, for each probe set, the median of all aCGH oligos located between the start and end base pair positions of the gene the probe set mapped to. Probe set to gene mappings were retrieved from NetAffx (April 2005) and all base pair positions are based on the May 2004 human genome build. The data have been deposited in NCBIs Gene Expression Omnibus (GEO, ) and are accessible through GEO Series record GSE15477.

### Selection and prioritization of genes from the NMD microarray data

The most promising candidate genes for harbouring truncating mutations based on the NMD data in each breast cancer cell line were selected using the following criteria: I) the gene was induced by emetine at least 1.5 fold (log2 scale) in the cancer cell line and not in any of the other cell lines. II) Expression in the clean (no emetine treatment) sample was lower than in any of the clean samples from all other cell lines. III) The gene was located in a heterozygously deleted region. IV) Expression after emetine treatment was over 50 units (to exclude genes expressed at levels which are not measured reliably).

### Patient cohorts

A previously described cohort of early stage breast cancer (I/II) [7,12] was used for validation. From this, DNA from 127 tumours was used to sequence the *RIC8A *gene. Gene expression data from 115 tumours of the same cohort were used for *in silico *validation of expression levels and 38 of these were validated by qRT-PCR. Methylation analysis was performed on 86 tumours from a cohort of early lesions of breast cancer, 27 pure DCIS, 27 pure invasive (< 15 mm) and 32 mixed lesions (invasive with *in situ *component) [13] and in 75 DNA tumour samples from patients with locally advanced breast cancer (T3/T4) [14]. Microarray data of *RIC8A *were further *in silico *validated in 251 primary breast tumours [6].

### Mutation analysis

PCR primers with M13 tails were designed to amplify the exons plus 50 bp of intronic sequence at both ends of each exon (for primer sequences see Additional file 1). PCR products were purified with PCR cleanup filter plates (Millipore) and sequenced using M13 primers and BigDye v.1.1 on a 3730 DNA analyzer (Applied Biosystems).

### cDNA synthesis and real-time PCR analysis

cDNA was generated in a total volume of 20 μl with 50 ng total RNA using GeneAmp^® ^Gold RNA PCR Core kit (Applied Biosystems). Real-time PCR experiments were performed using TaqMan Gene Expression Assays (Applied Biosystems) on an ABI Prism 7900 HT sequence detector system (Applied Biosystems). The primers included were designed to overlap exon-exon boundaries to avoid genomic DNA contamination. Universal human reference RNA (Stratagene) was used to generate standard curves. Each sample was run in triplicate and the relative gene expression levels were determined with the standard curve method and normalized using *PGK1 *as reference gene.

### DNA methylation analysis by pyrosequencing

1 μg of DNA was bisulphite converted using the MethylEasy™ HT Kit for Centrifuge (Human Genetic Signatures, North Ryde, Australia) according to the manufacturer's instructions. Quantitative DNA methylation analysis of the bisulphite treated DNA was performed by pyrosequencing [15]. 98 CpGs spanning 2 kb of genomic sequence were quantitatively analyzed at single nucleotide resolution. The investigated regions comprised the entire promoter region as well as other regions of the large CpG island (nucleotides -943 to +1338 relative to the transcription start site). Oligonucleotides for PCR amplification and pyrosequencing were synthesized by Biotez (Buch, Germany) and sequences are given in Additional file 2.

### Statistics

Student's t-test and Mann-Whitney test were performed using SPSS (version 15.0. SPSS Inc, Chicago, Illinois, USA) for association with clinical parameters. In the microarray datasets used for validation, samples with *RIC8A *signal intensity < 650 were considered as low expressed and *RIC8A *signal intensity > 950 as highly expressed. With this range, about 15% of the tumours showed low expression, a frequency similar to what was observed in other published data sets [16,17].

## Results

### Identification of genes subject to NMD-degradation by microarray analysis

**Figure 2:**
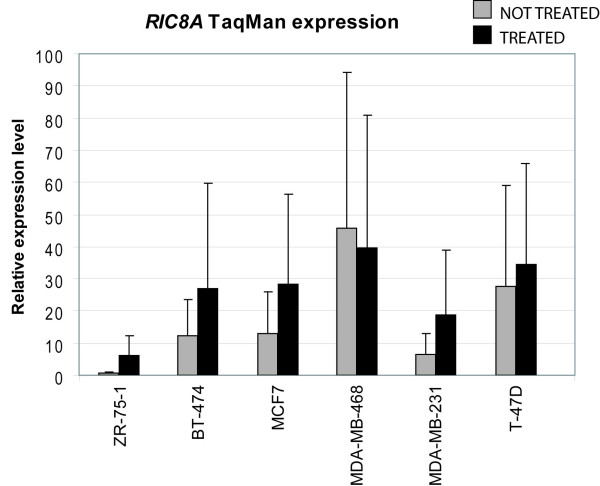
**Validation of *RIC8A *expression in cell lines by qRT-PCR**. Relative expression level of *RIC8A *measured by TaqMan qRT-PCR in treated vs. non-treated cell lines is shown with black and grey bars, respectively. Each sample was run in triplicate and the relative gene expression levels were determined with the standard curve method and normalized using *PGK1 *as reference gene.

We measured levels of gene expression by microarrays before and after pharmacological inhibition of the NMD-pathway in order to identify candidate genes for mutation screening in six breast cancer cell lines (MDA-MB-468, MDA-MB-231, ZR-75-1, MCF7, BT-474 and T-47D). In total, using our prioritization criteria (see Methods) for the NMD microarray results, 51 candidate genes were identified of which three were prioritized for analysis by DNA sequencing to identify putative mutations (Additional file 3). The normalized NMD microarray ratios for the three genes were 1) 1.89 for *PGPEP1 *(Entrez GeneID:54858) in the BT-474 cell line compared to the other five cell lines, 2) 5.16 for *COL12A1 *(Entrez GeneID:1303) in ZR-75-1 cells compared to the other lines, and 3) 1.5 for *RIC8A *(Entrez GeneID:60626) in ZR-75-1 cells, also compared to the other five cell lines. The three selected candidate genes were sequenced in all six breast cancer cell lines. Sequence analysis of *RIC8A *confirmed a hemizygous nonsense mutation, GAG → TAG in the last codon of exon 3 in the ZR-75-1 cell line, and not in any of the other cell lines. Further, *RIC8A*, located at 11p15.5, was heterozygously deleted according to the aCGH data and showed a low level of expression also in non-treated ZR-75-1 cells compared to the other cell lines, suggesting bi-allelic inactivation of the gene in these cells (Figure 1A+B). We confirmed the differential expression of *RIC8A *in the ZR-75-1 cell line compared to the other cell lines by qRT-PCR (Figure 2). The remaining two candidate genes selected for sequencing displayed wild type DNA sequences in all six breast cancer cell lines.

### Sequencing of *RIC8A *in clinical breast cancer samples

To further determine the relevance of the mutation in clinical specimens, we sequenced all ten exons of *RIC8A *in 127 tumour samples obtained from patients with early-stage breast cancer. We could not find nonsense mutations or other amino acid changing mutations in any of these tumours. Notably, we have previously demonstrated the ability to detect mutations in only 10–15% of cells in a sample with this sequencing technology.

To evaluate if *RIC8A *could be silenced by CpG hypermethylation in the promoter regions, we pyrosequenced 98 CpGs spanning 1 kb upstream of the transcription start site as well as the 3' end of the large CpG island downstream of the transcription start site in pre-invasive (DCIS) (n = 27) and invasive tumours with DCIS components (n = 32), in early invasive breast cancer (n = 27) as well as a cohort of 75 locally advanced breast cancers. All CpG positions upstream of the transcription start were found to be unmethylated in all samples, while some methylation was observed at CpGs downstream of the transcription start as well as at the 3' end of the CpG island where methylation levels reached 40–80%. However, DNA from normal breast tissue samples displayed identical methylation patterns indicating that *RIC8A *is not a target for epigenetic silencing (data not shown).

### Bioinformatics and qRT-PCR analysis of *RIC8A *in clinical breast cancer cohorts

**Figure 3:**
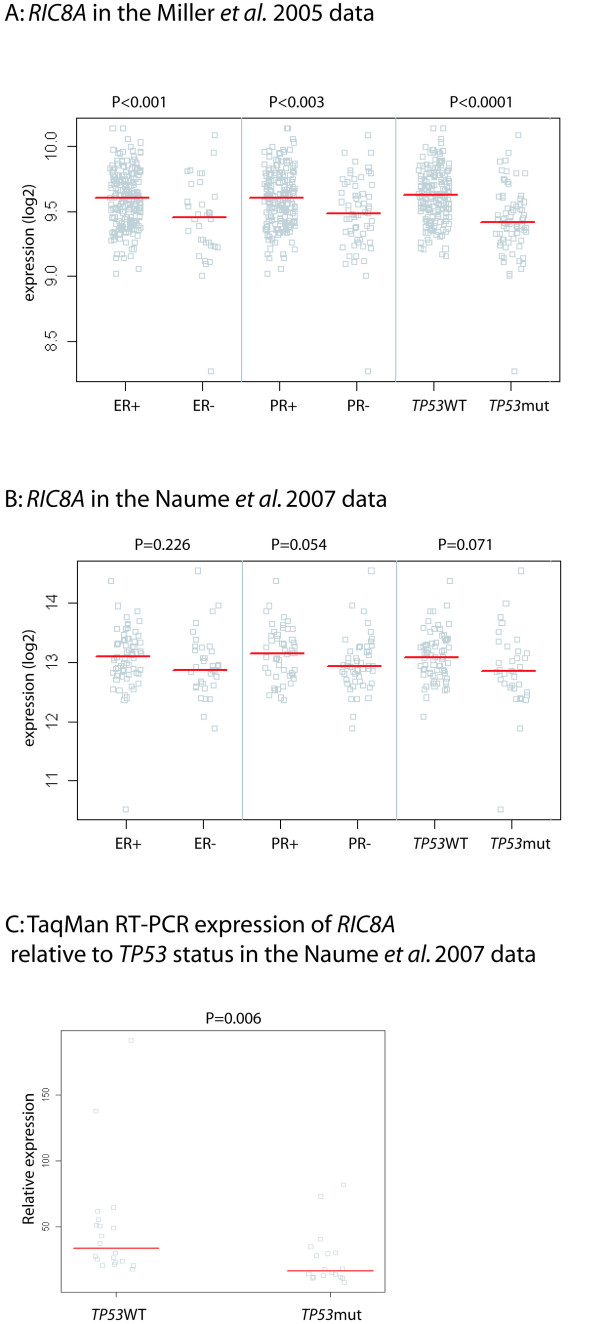
***RIC8A *expression levels associated with different molecular parameters**. A: Gene expression in the Miller *et al*. 2005 data [6]: The 251 breast tumours separated based on ER, PR and *TP53 *status. *TP53 *mutated group includes all tumours with a signature of *TP53 *inactivation [6]. B: Gene expression in the Naume *et al*. 2007 data [7]: The 115 breast tumours separated based on ER, PR and *TP53 *status. C: *RIC8A *expression by TaqMan qRT-PCR. The 38 tumours separated based on *TP53 *mutation status. Each square represents the expression level of *RIC8A *in one tumour. Red bars indicate group medians.

To examine the importance of *RIC8A *as a target gene for the frequent deletion in chromosome band 11p15 in breast cancer, we analyzed the expression of *RIC8A *in previously published breast cancer microarray data sets. First, a gene expression dataset consisting of 251 consecutively collected breast cancers [6] displayed low *RIC8A *transcript levels in 15% of the tumours and a significant association was shown between low *RIC8A *gene expression and ER-negativity (P < 0.001), PR-negativity (P < 0.003) and *TP53 *mutation (P < 0.0001) (Figure 3A). Second, a smaller dataset of 115 early-stage breast tumours [7] displayed low *RIC8A *expression in 19% of the tumours and showed borderline significant association between low *RIC8A *gene expression and PR-negativity (P = 0.054) and *TP53 *mutation (P = 0.071) (Figure 3B). Finally, qRT-PCR analysis by TaqMan of 38 of these tumours validated that there was an association between low *RIC8A*- expressing tumours and the presence of *TP53 *mutations (Mann-Whitney test: P = 0.006) (Figure 3C).

## Discussion

We describe here an integrated genomic approach for the genome-wide-screening for putative tumour suppressor genes in cancer. Given the multitude of potential target genes in frequently deleted regions of the cancer genome, strategies to narrow down the candidates for detailed study would lead to more efficient identification of cancer genes. Here, we describe how a data integration approach combining three microarray technologies (aCGH, gene expression arrays and NMD microarrays) made it possible to narrow the search for potential mutated genes to three tumour suppressor candidates, one of which, *RIC8A*, was confirmed to show a truncating mutation, deletion and loss of expression in a breast cancer cell line. Furthermore, while the clinical breast cancers had no clear pathogenetic mutations, a subset of clinical breast cancers associated with aggressive features showed low levels of *RIC8A *expression indicating that inactivation of this gene may play a role in the pathogenesis of a subset of breast cancers.

This represents the second example of NMD-based discovery identifying a novel truncating mutation in cancer. Our previous studies of prostate cancer cell lines identified *EPHB2 *as a target for similar deletion-truncation events in the DU-145 cells [3]. Therefore, it is clear that some, but not all mutations are highlighted by the NMD-based strategy, particularly in combination with the aCGH data. Treatment with translation inhibitors, such as emetine, extends the half-lives of numerous transcripts [18]. In addition, nonsense-mediated mRNA decay (NMD) is known to regulate a significant number of genes under physiological conditions [19], as well as to prevent incorrectly spliced genes from being translated [20]. To avoid selection of stress-response genes or those normally regulated by NMD, we applied stringent filtering criteria to the NMD microarray data from six breast cancer cell lines. These included first, presence of the candidate gene in a deleted region of the genome in that cell line, corresponding with the two-hit Knudson hypothesis of tumour suppressor gene inactivation, and second, a low relative baseline mRNA expression. Using this integrated genomic approach we limited the list of candidates for sequencing to 51. The three top candidate genes with the clearest increase in normalized NMD ratios were sequenced in all cell lines. Of these, *RIC8A *showed a truncating mutation.

After discovery of a mutation in a cancer cell line, the more challenging task is to ascertain the significance and prevalence of such mutations in clinical samples. The key question here is as to whether the mutation is critical to the pathogenesis of cancer and common across a set of cancers, or possibly a "passenger mutation" selected along with a more important other mutation. Although mutations in the coding region of *RIC8A *were not found in the cohort of 127 early-stage breast cancers, we cannot rule out that mutations might exist at a low frequency in more advanced cases. Exhaustive pyrosequencing of the entire *RIC8A *promoter region in early invasive breast cancers as well as in pre-invasive tumours (DCIS) and locally advanced (T3/T4) tumours excluded also promoter hypermethylation as a mechanism for *RIC8A *gene silencing. Recent estimates of the prevalence of somatic mutations in human cancers are highly variable both within and between classes of cancer [21]. The emerging pattern is that there are relatively few frequently mutated genes, (most of these already known) and a very large number of rare mutations, often highly varying from one case to another [22]. Based on the lack of mutations identified in clinical breast cancer samples as well as the lack of evidence from cancer genome sequencing studies indicating involvement of *RIC8A *[22,23], we postulated that *RIC8A *inactivation could either be a rare genetic event in breast cancer, or possibly, its inactivation in clinical samples could take place more often through other mechanisms, not involving direct truncating mutations or methylation.

*RIC8A *was originally found by genetic studies in *C. elegans *and reported to act *in vitro *as a guanine nucleotide exchange factor (GEF) for G protein alpha subunits [8]. Several studies have since reported that *RIC8A *is required for G-protein signalling and is involved in centrosome movements during early embryogenesis in *C. elegans *[24,25]. In addition, *RIC8A *was discovered to be required for proper asymmetric division of one-cell-stage in both *C. elegans *embryos [26] and *Drosophila *[9]. However, the physiological role of the mammalian homolog *RIC8A *in G protein-coupled receptor signalling in intact cells is largely unknown. Yet, a human mitotic phosphorylation motif associated with protein localization to the mitotic apparatus was recently identified in *RIC8A*, suggesting a role for *RIC8A *in mitosis [10]. This point to the possibility that *RIC8A *would play a role in cancer relevant pathways and that its inactivation could be important to the pathogenesis of the disease.

Bioinformatics analysis of microarray data from 251 consecutive breast cancers [6] showed that low *RIC8A *expression was significantly associated with hormone receptor negativity (ER and PR) as well as presence of *TP53 *mutations. A similar analysis of gene expression in 115 tumours from another cohort focussing on early breast cancer indicated a similar, but statistically weaker trend (Figure 3). The association between low *RIC8A *expression and *TP53 *mutations was validated by the analysis using qRT-PCR of 38 tumours from the early stage cohort [7]. Furthermore, a previously published analysis of 171 primary breast tumours showed that the chromosome 11p telomeric region is lost at an approximate frequency of 15% [16]. The frequency is highest towards the distal part of the chromosome, where *RIC8A *is located, and declines towards the centromere. In the same tumours, genes in this region, including *RIC8A*, were shown to be statistically significantly deleted and underexpressed when combining copy number and gene expression data. Also, this deletion was statistically significantly associated to both recurrence and distant metastases in this patient cohort. There was no significant association with any other clinical parameter. Yet another study showed that 11p15.5-p15.4 copy number loss in primary breast cancers was associated with a higher incidence of recurrent disease in tamoxifen-treated patients [17]. While *RIC8A *is located in one of the most frequently deleted regions in breast cancer [27], the deletion obviously involves a large number of genes. We therefore evaluated whether the flanking genes from *RIC8A *would show similar statistical associations between low expression and ER/PR negativity and *TP53 *mutation, respectively. For example, this region harbours the *SIRT3 *gene, which has been associated with cancer [28,29] and whose expression showed correlation with copy number loss together with *RIC8A *[16]. However, in our analyses *RIC8A *showed the strongest association with aggressiveness. Together with the presence of the truncating mutation, this suggests a specific importance of the *RIC8A *gene in a subset of breast cancers.

## Conclusion

We demonstrate here that integrating microarray data from three different methods (aCGH, expression, nonsense-mediated mRNA decay) facilitated the identification of putative tumour suppressor genes undergoing bi-allelic inactivation in cancer. Here, this strategy led to the identification of *RIC8A *as a gene undergoing deletional and mutational inactivation in a breast cancer cell line, as well as *in vivo *evidence of loss of its expression in a subgroup of aggressive breast cancers.

## Competing interests

The authors declare that they have no competing interests.

## Authors' contributions

AAM performed the laboratory experiments, analyses of data and writing the manuscript.

HE performed the data analyses and writing the manuscript. MW was involved in study design, laboratory experiments, analyses of data and writing the manuscript. KK was involved in study design, analyses of data and writing the manuscript. ED and JT were involved in the methylation analyses. TS and OK initiated and designed the study and were involved in writing the manuscript. All authors read and approved the final manuscript.

## Pre-publication history

The pre-publication history for this paper can be accessed here:



## Supplementary Material

Additional file 1 **DNA sequencing primers.** Click here for file

Additional file 2 **Pyrosequencing primers.** Click here for file

Additional file 3 **List of the 51 candidate genes selected for sequencing.** Click here for file
